# A127 ENDOSCOPIC MUCOSAL RESECTION WITH HYBRID-ARGON PLASMA ABLATION TO PREVENT RECURRENCE OF 20 MM NON-PEDUNCULATED COLORECTAL POLYPS: A MULTI-CENTER PROSPECTIVE CLINICAL STUDY

**DOI:** 10.1093/jcag/gwad061.127

**Published:** 2024-02-14

**Authors:** R Djinbachian, J M Levenick, S Bouchard, M T Moyer, E Deslandres, J Mosko, C Teshima, N Shahidi, D von Renteln

**Affiliations:** CRCHUM, Montréal, QC, Canada; Penn State Hershey Medical Center and Penn State College of Medicine, Hershey, PA; CRCHUM, Montréal, QC, Canada; Penn State Hershey Medical Center and Penn State College of Medicine, Hershey, PA; CRCHUM, Montréal, QC, Canada; The Centre for Therapeutic Endoscopy and Endoscopic Oncology, St. Michael's Hospital, University of Toronto, Toronto, ON, Canada; The Centre for Therapeutic Endoscopy and Endoscopic Oncology, St. Michael's Hospital, University of Toronto, Toronto, ON, Canada; Department of Medicine, University of British Columbia, Vancouver, BC, Canada; CRCHUM, Montréal, QC, Canada

## Abstract

**Background:**

Endoscopic mucosal resection (EMR) is the mainstay of therapy for non-pedunculated colorectal polyps 20 mm or larger. Post EMR recurrence rates are about 15% if no margin ablation technique is used.

**Aims:**

Study aim was to evaluate recurrence rates if hybrid Argon Plasma Coagulation ablation (h-APC) is routinely used after the EMR to ablate the margins, base and vessels.

**Methods:**

A prospective multi-center study including adult patients (18-89 years) undergoing EMR of non-pedunculated colorectal polyps 20 mm or larger was conducted. h-APC was used to ablate all post EMR margins and to ablate the post EMR resection surface and visible submucosal vessels. The primary outcome was the biopsy proven recurrence rate at the first follow-up colonoscopy (4-6 months after EMR). All the resection sites were visually inspected, and biopsies were obtained from the EMR scar. Secondary outcomes included technical success and complication rates.

**Results:**

A total of 220 EMRs were performed during the study for h-APC ablation of non-pedunculated colorectal polyps, with a size of ≥20 mm. The average size of the polyps included in the study was 35.7 mm (ranging from 20 mm to 100 mm). Among all resected polyps, 16.8% were sessile serrated lesions (SSL), while 66.3% were adenomas. Additionally, 4 cases of cancer were in the study. The application of hot snare EMR with h-APC ablation was technically successful and completed for all cases. The mean duration of the EMR procedure was 19.6 minutes, ranging from 2.3 to 103 minutes, while the mean duration of h-APC ablation was 6.4 minutes, ranging from 1 to 65 minutes.

A total of 147 EMRs with follow up examination (57 EMR involving lesions 40mm or larger) are available currently. The overall recurrence rate was 1.3% (2 out of 147 cases). For cases where complete base ablation was achieved, there was a 0% recurrence rate, including lesions 40mm or larger. For lesions measuring 40mm or larger, the recurrence rate was 1.8% (1 out of 54 cases). Out of the 220 total EMRs conducted, perforations were observed in 0.9% (2 cases, managed conservatively with antibiotics only), and clinically significant bleeding occurred in 2.3% (5 cases). In particular, clinically significant bleeding was noted in 2.3% (4 out of 167 cases) of lesions without clipping and in 4.0% (4 out of 99 cases) for right-sided lesions without clipping.

**Conclusions:**

In this multicenter study EMR in combination with h-APC demonstrated a high technical success rate with low complication rates and showed very low post EMR recurrence in the preliminary data analysis.

**Funding Agencies:**

Erbe

